# High-resolution magnetic resonance imaging-based radiomic features aid in selecting endovascular candidates among patients with cerebral venous sinus thrombosis

**DOI:** 10.1186/s12959-023-00558-4

**Published:** 2023-11-10

**Authors:** Yu-zhou Chang, Hao-Yu Zhu, Yu-Qi Song, Xu Tong, Xiao-Qing Li, Yi-Long Wang, Ke-Hui Dong, Chu-Han Jiang, Yu-Peng Zhang, Da-Peng Mo

**Affiliations:** 1 Department of Interventional Neuroradiology, Beijing Neurosurgical Institute, Beijing Tiantan Hospital, Capital Medical University, No.119 South 4th Ring West Road, Beijing, Fengtai District 100070 P.R. China; 2 Department of Neurosurgery, Beijing Tiantan Hospital, Capital Medical University, Beijing, China; 3 Interventional Neuroradiology Center, Department of Neurology, Beijing Tiantan Hospital, Capital Medical University, Beijing, China; 4 Department of Neurology, Beijing Tiantan Hospital, Capital Medical University, Beijing, China

## Abstract

**Objectives:**

Cerebral venous sinus thrombosis (CVST) can cause sinus obstruction and stenosis, with potentially fatal consequences. High-resolution magnetic resonance imaging (HRMRI) can diagnose CVST qualitatively, although quantitative screening methods are lacking for patients refractory to anticoagulation therapy and who may benefit from endovascular treatment (EVT). Thus, in this study, we used radiomic features (RFs) extracted from HRMRI to build machine learning models to predict response to drug therapy and determine the appropriateness of EVT.

**Materials and methods:**

RFs were extracted from three-dimensional T1-weighted motion-sensitized driven equilibrium (MSDE), T2-weighted MSDE, T1-contrast, and T1-contrast MSDE sequences to build radiomic signatures and support vector machine (SVM) models for predicting the efficacy of standard drug therapy and the necessity of EVT.

**Results:**

We retrospectively included 53 patients with CVST in a prospective cohort study, among whom 14 underwent EVT after standard drug therapy failed. Thirteen RFs were selected to construct the RF signature and CVST-SVM models. In the validation dataset, the sensitivity, specificity, and area under the curve performance for the RF signature model were 0.833, 0.937, and 0.977, respectively. The radiomic score was correlated with days from symptom onset, history of dyslipidemia, smoking, fibrin degradation product, and D-dimer levels. The sensitivity, specificity, and area under the curve for the CVST-SVM model in the validation set were 0.917, 0.969, and 0.992, respectively.

**Conclusions:**

The CVST-SVM model trained with RFs extracted from HRMRI outperformed the RF signature model and could aid physicians in predicting patient responses to drug treatment and identifying those who may require EVT.

**Supplementary Information:**

The online version contains supplementary material available at 10.1186/s12959-023-00558-4.

## Introduction

Cerebral venous sinus thrombosis (CVST) refers to obstruction of the venous outflow channel after thrombosis formation, resulting in cerebral venous sinus hypertension, impaired cerebral venous drainage, and cerebrospinal fluid absorption [1]. A recent increase in the incidence of CVST has led to increased attention from neurologists and neurosurgeons [2]. The annual incidence of CVST is 2–5 per 10 million people, accounting for approximately 1% of all stroke cases. Although anticoagulation drug therapy is the standard first-line treatment for CVST [3], such treatments may fail for patients with large and dense thromboses because these types of thrombosis can increase the difficulty of drug penetration, thereby reducing treatment efficacy [4]. Endovascular treatment (EVT) has emerged as an effective second-line treatment option for patients who show no improvement after anticoagulation therapy. Because patients who require endovascular treatment are usually accompanied with more serious complications, intracranial hemorrhage or brain herniation during or after surgery will lead to patient death. Thus, more dependable methods are needed to identify appropriate candidates for initiating mechanical thrombectomy before drug treatment failure is observed [5].

Magnetic resonance imaging (MRI) is a promising non-invasive technique for CVST diagnosis. Previous studies have combined T1-weighted and T2-weighted sequences to estimate the temporal dynamics of the thrombus [6, 7]. Black-blood MRI can also be performed to analyze the signal intensity of the thrombus and vessel wall, providing insight into the degree of recanalization. Motion-sensitized driven equilibrium (MSDE) turbo spin-echo (TSE) sequences obtained using high-resolution MRI (HRMRI) can be used to generate black-blood images that allow for the extraction of even more radiomic features (RFs) to describe the characteristics of tissues and lesions, which can aid in evaluating thrombus characteristics such as texture and uniformity. These analyses may help stratify patients more appropriately, enabling personalized treatment plans to be developed [8].

Thus, in this study, we extracted RFs from all HRMRI sequences that all 53 patients completed (including T1-weighted-MSDE, T2-weighted-MSDE, T1-weighted-contrast-MSDE, and T1-weighted-contrast images). The HRMRI provided finer image details than the previous study which provided more delicate information. Lasso regression and support vector machine method, which were better applied to small sample sizes, were capable of predicting the efficacy of drug therapy and identifying CVST patients who may require EVT. Furthermore, we clarified the relationship between machine learning models and clinical characteristics to figure out that the models could describe the clinical manifestation as well. In short, the machine learning models based on the radiomics features extracted from the high-resolution MRI were established to evaluate the candidate CVST patients that need and benefit from the EVT.

## Methods

### Patient enrollment

The ethical committee of Beijing Tiantan Hospital (KY2016-039-02) approved this study, and all patients provided written informed consent.

**Fig. 1:**
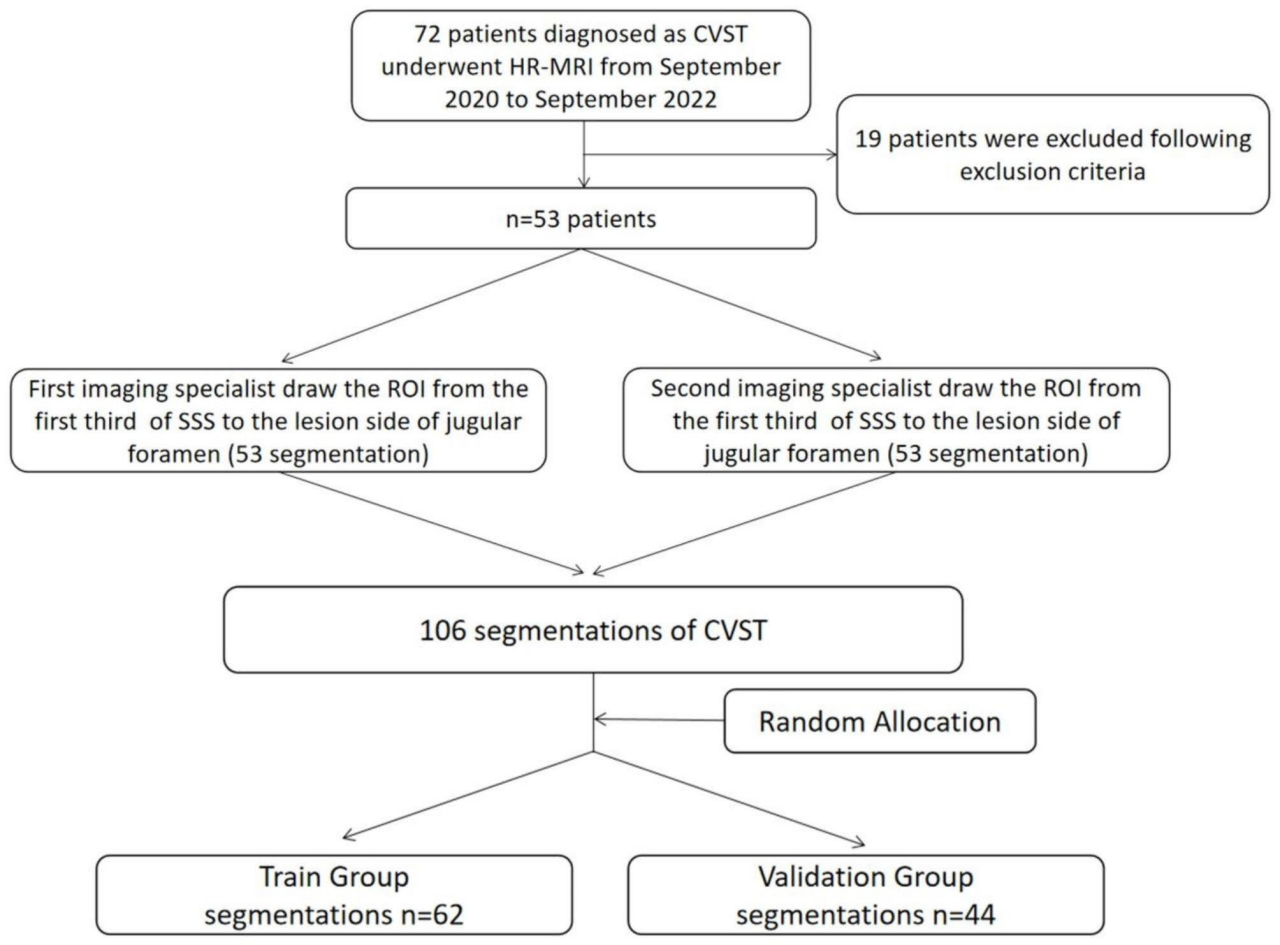
Flowchart with the inclusion and exclusion criteria: 72 patients diagnosed as CVST underwent HRMRI from September 2020 to September 2022. 19 patients were excluded following exclusion criteria. Two experienced neuroradiologists independently draw the RIO from the HRMRI sequences. All 106 segmentations were randomly separated into the training group and the validation group for further analysis

For our prospective CVST cohort, we retrospectively included 53 of 72 patients diagnosed with CVST who underwent HRMRI before standard drug treatment at our institution between September 2020 and September 2022 (Fig. 1). The included patients met the following criteria: (1) availability of high-quality HRMRI data before initiating medical treatment and (2) receipt of standard treatment by the Chinese Stroke Association guidelines [1]. The exclusion criteria were as follows: (1) absolute contraindication to EVT, (2) failure to achieve recanalization after endovascular mechanical thrombectomy, and (3) loss to follow-up or recurrence of thrombosis during the study period. The outcomes of patients receiving the drug and combined treatment were assessed based on main symptoms and the modified Rankin Scale (mRS) [9]. Based on the aims of this study, a good outcome was defined as achieving an mRS score ≤ 2 and experiencing [10] relief from the main symptoms. All patients were followed up for 1 month after discharge.

### CVST treatment

Patients diagnosed as having CVST were admitted, and all of them received drug treatment. Low-molecular-weight heparin (LMWH) or nadroparin (0.6 ml) was subcutaneously injected twice a day at treatment initiation, with treatment lasting for 1–4 weeks. Oral administration of warfarin was used to maintain an international normalized ratio of 2–3 that lasted for 3–6 months. Patients with concurrent intracranial hemorrhage were treated with rivaroxaban (15–20 mg daily) instead of warfarin. Failure of conservative treatment was defined as the absence of relief or deterioration of symptoms after 3–5 days of drug treatment. These patients underwent EVT for further treatment.

For EVT, we selected the aspiration-first strategy for all patients. An 8-F guiding catheter was placed at the jugular bulb level, and a 6-F intermediate catheter (132 cm Catalyst, Stryker, Fremont, CA) was navigated to the superior sagittal sinus (SSS) over a 260-cm glidewire, which macerated the thrombosis. Next, we switched to a 300-cm 0.014-inch Command microwire (Abbott, Chicago, IL, USA) and introduced a 4-mm × 30-mm-sized balloon into the SSS. Using a combination of balloon dilation and aspiration via the Catalyst, we could remove the thrombosis in a distal-to-proximal direction. After thrombectomy, a 0.027-inch microcatheter was left in the SSS to facilitate continuous delivery of urokinase for 3–5 days at a dose of 25,000–50,000 IU/h.

### Image acquisition

All images were acquired using a 3.0-Tesla system (Ingenia CX, Philips Healthcare, Best, The Netherlands) equipped with a 32-channel head coil. T1-weighted-MSDE, T2-weighted-MSDE, T1-weighted-contrast-MSDE, and T1-weighted-contrast images were used to delineate the lesion and extract the location and RFs. T1-weighted-MSDE image parameters were as follows: repetition time, 800 ms; echo time, 21.081 ms; flip angle, 90°; 228 slices; voxel size, 0.681 × 0.681 × 0.7 mm^3^; acquisition matrix, 344 × 343; scanning technique, TSE; and pixel bandwidth, 349 Hz/pixel. T2-weighted-MSDE parameters were as follows: repetition time 2,500 ms; echo time, 190.358 ms; flip angle, 90°; 229 slices; voxel size, 0.681 × 0.681 × 0.7 mm^3^; acquisition matrix, 344 × 342; scanning technique, TSE; and pixel bandwidth, 349 Hz/pixel. T1-weighted-contrast-MSDE image parameters were as follows: repetition time, 800 ms; echo time, 21.453 ms; flip angle, 90°; 228 slices; voxel size 0.681 × 0.681 × 0.7 mm^3^; acquisition matrix, 344 × 343; scanning technique, TSE; and pixel bandwidth, 349 Hz/pixel. T1-weighted-contrast image parameters were as follows: repetition time, 6.526 ms; echo time, 2.998 ms; flip angle, 8°; 196 slices; voxel size, 1.0 × 1.0 × 1.0 mm^3^; acquisition matrix, 240 × 240; scanning technique, TSE; and pixel bandwidth, 241 Hz/pixel.

### RF extraction

Segmentation of the region of interest (ROI) started from the anterior third of the SSS, extending along the transverse sinus and sigmoid sinus with thrombosis, to the end of the internal jugular vein. Two experienced neuroradiologists who were blinded to each other used the open-source software 3D Slicer (https://www.slicer.org) to delineate ROIs on the 3D T1-contrast image. To include features in other sequences, the T1-weighted-MSDE, T2-weighted-MSDE, and T1-contrast-MSDE images were co-registered to the 3D T1-contrast image with SPM12, and all voxels of the images were resampled to 1.0 × 1.0 × 1.0 mm^3^. A total of 1,274 RFs were extracted from each sequence using the “PyRadiomics” package in Python (3.6.4) [11]. Among the RFs, there were 18 first-order statistic features, 22 Gy-level co-occurrence matrix texture features, 16 Gy-level run-length matrix texture features, 16 Gy-level size zone matrix (GLSZM) texture features, 14 Gy-level dependence matrix texture features, and 5 neighboring gray-tone difference matrix features. In total, there were 91 original filter features, 455 Laplacian of Gaussian filter image features, and 728 wavelet filter features.

### Construction of the RF signature model

RFs were used to construct a signature model (RF signature model). To increase the sample size, we combined all segmentations drawn by the neuroradiologists. All segmentations were randomly separated into training and validation groups at a ratio of 6:4. In the training group, the least absolute shrinkage and selection operator (LASSO) with three-fold cross-validation was used to select informative features [12]. Then, we calculated the RF signature as follows:

RF signature = ∑feature values × Cox efficient of feature.

The performance of the RF signature in discriminating EVT was tested in the training and validation datasets using sensitivity, specificity, accuracy, and F1-score. The optimal cutoff point was determined using the Youden index. Finally, the relationship between the RF signature model and clinical characteristics was also examined.

### Construction of the support vector machine (SVM) model

The features extracted from the LASSO regression were further used to build an SVM model (CVST-SVM). A grid search method was performed to tune the hyperparameters of the SVM. The gamma value and cost parameter C were tested at 0.0001, 0.001, 0.01, 0.1, 1, 10, and 100 [13]. The prediction accuracy was maximized using the best combination of gamma and parameter C. To further evaluate the CVST-SVM model, the mean variable importance of over 1,000 permutations was used to rank the explanatory importance of the included RFs.

### Statistical analyses

Statistical analyses were performed using R v3.6.1 (R Foundation for Statistical Computing, Vienna, Austria), SPSS 26.0 (SPSS, Inc., Chicago, IL, USA), and Prism 8 (GraphPad Software, Inc., La Jolla, CA, USA). The chi-square test was performed to assess the distribution of clinical characteristics between the two treatment groups. Two-tailed Student’s t-test was performed to compare risk scores in patients grouped on the basis of other clinical characteristics or stratified by risk score. Moreover, two-tailed Student’s t-test was performed to evaluate the top three features in the CVST-SVM model in the two treatment groups. *P*-values < 0.05 were considered statistically significant in all analyses.

## Results

### Baseline characteristics

This study included 53 patients with CVST who had high-quality HRMRI data (Table 1). Among them, 39 (73.5%) patients were treated with anticoagulant therapy only and 14 (26.4%) were treated with anticoagulant therapy combined with salvage mechanical thrombectomy. Smoking history was significantly more common in the combined treatment group than in the anticoagulant-only group (*p* < 0.01), although there were no significant differences in other clinical characteristics such as age, sex, body mass index (BMI), days from symptom onset, number of venous sinuses involved, related medical history, and accompanying symptoms. Given that previous studies have reported close associations between CVST and coagulation function, we also assessed coagulation-related indices and platelet counts. There were no significant differences in coagulation function between the two groups (Table 1).

**Table 1:** Baseline characteristics of patients in different treatment groups

Variable	Drug treatment (n = 39)	Combined treatment (n = 14)	*p*-value
**Age (years; median)**	39 (13–68)	34 (23–62)	**0.48**
**Sex (female) (number)**	16 (41%)	5 (35.7%)	**0.73**
**BMI (median)**	25.7 (18.3–37.2)	27.1 (19.8–51.4)	**0.49**
**Date from symptom onset (median) (day)**	10 (1–365)	17 (2–100)	**0.38**
**Venous sinus involved (> 1 segment)**	33 (84.6%)	14 (100%)	**0.12**
**History of cysteinemia (number)**	12 (30.7%)	5 (35.7%)	**0.73**
**History of contraceptive usage (number)**	3 (7.7%)	1 (7.1%)	**0.95**
**History of hypertension (number)**	9 (23%)	2 (14.3%)	**0.49**
**History of dyslipidemia (number)**	16 (41%)	7 (50%)	**0.56**
**History of blood coagulation disfunction (number)**	14 (35.9%)	5 (35.7%)	**0.99**
**Smoking (number)**	8 (20.5%)	9 (64.3%)	0.003*
**Accompany with cerebral hemorrhage (number)**	11 (28.2%)	4 (28.6%)	**0.98**
**Accompany with cerebral venous infarction (number)**	9 (23.1%)	4 (28.6%)	**0.19**
**ICP (> 200 mmH**_**2**_ **O) (number)**	19 (61.3%)	10 (90.9%)	**0.09**
**Coagulation function (proper value)**			
**Fibrin degradation product (µg/mL)**	2.84 (0.34–15.13)	2.3 (0.7–19.2)	**0.41**
**D-Dimer (µg/mL)**	1 (0.05–9.88)	1.04 (0.39–10.38)	**0.82**
**Prothrombin time (INR)**	1.08 (0.92–3.73)	1 (0.92–4.37)	**0.22**
**Activated partial thromboplastin time (s)**	29.6 (16.6–54.4)	28.5 (24.4–46.5)	**0.23**
**Thrombin time (s)**	14 (11.5–17.8)	13.9 (11.7–16.6)	**0.65**
**Fibrinogen (g/L)**	3.48 (2.09–5.47)	3.47 (2.14–4.87)	**0.6**
**Platelet (10** ^**9**^ **/L)**	244 (99–612)	232 (180–345)	**0.33**
**Platelet distribution width (fL)**	15.9 (15.3–17.2)	16 (15.5–16.5)	**0.67**
**Mean platelet volume (fL)**	9.1 (7.3–10.5)	8.7 (7.7–10.6)	**0.15**
**Platelet - larger cell ratio**	20.4 (9.4–30.6)	16.7 (11.2–31.6)	**0.54**

BMI: body mass index; ICP: intracranial hypertension; INR: international normalized ratio

### RFs

Two professional neuroradiologists delineated the ROIs in each patient, and RFs were extracted from 3D T1-weighted-MSDE, T2-weighted-MSDE, T1-contrast, and T1-contrast-MSDE MR images. A total of 5,096 RFs extracted from the venous sinus ROIs were used for further analysis. The two ROIs drawn by different neuroradiologists were mixed to generate a dataset of 106 segmentations, which were randomly split into a training (n = 62) and validation group (n = 44) at a ratio of 6:4.

### Performance of the RF signature model

**Fig. 2:**
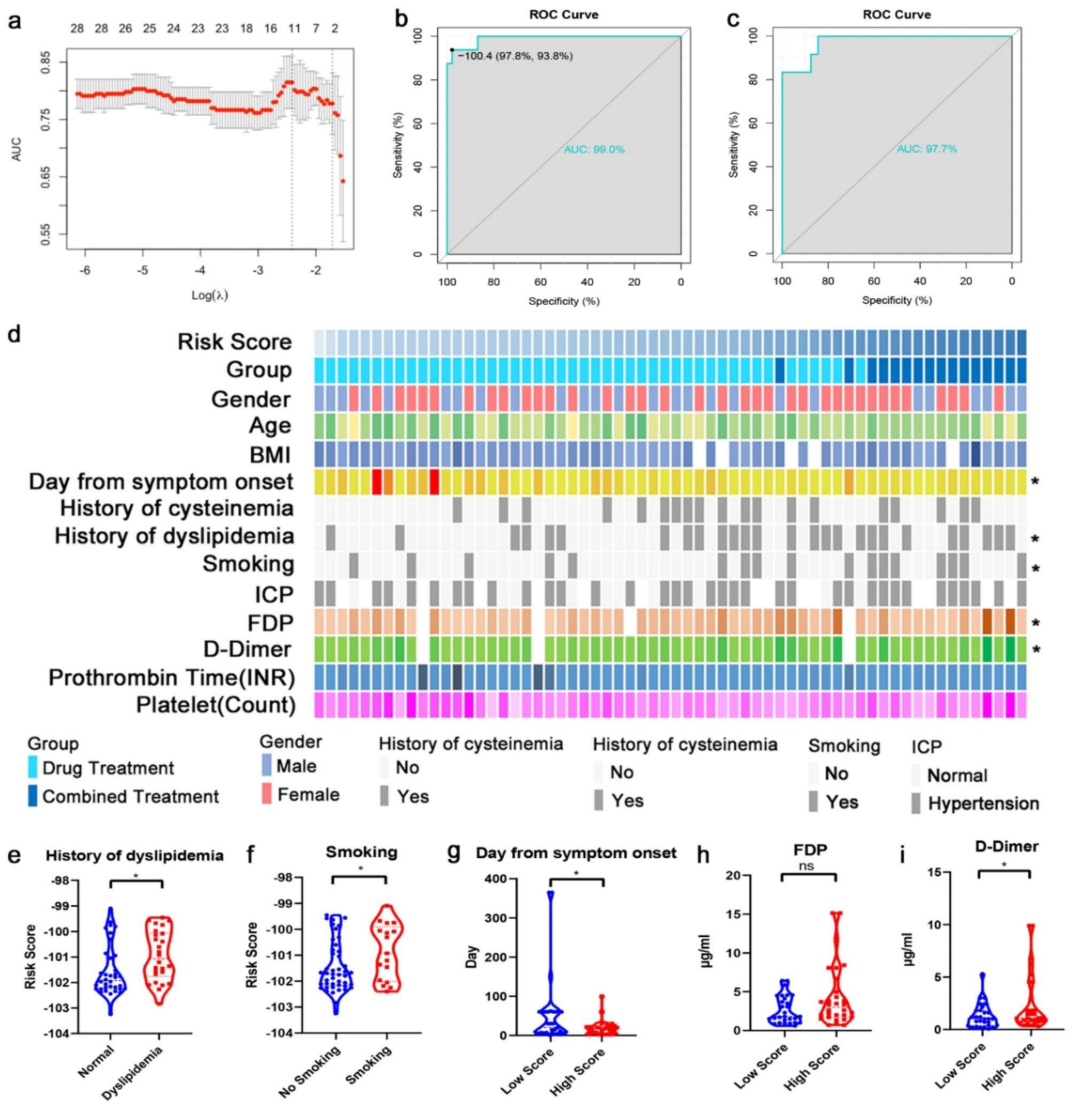
Performance of the radiomic signature model. (**a**) A total of 13 RFs were selected based on least absolute shrinkage and selection operator (LASSO) regression. (**b**) The receiver operating characteristic (ROC) curve of the signature model in the training group. The optimal cutoff of the signature model was − 100.4. (**c**) The ROC curve of the signature model in the validation group. -100.4 was used as the optimal cutoff. (**d**) The distribution of clinic characteristics arranged according to increasing risk scores. (*, p < 0.05) (**e**–**f**) The distribution of risk scores stratified according to history of dyslipidemia and smoking. (**g**–**i**) The distribution of days from symptom onset, fibrin degradation product, and D-dimer levels stratified according to risk scores (*, p < 0.05). AUC: area under curve; BMI: body mass index; FDP: fibrin degradation product; ICP: intracranial hypertension; INR: international normalized ratio; ROC: receiver operating characteristic

Thirteen features were extracted using LASSO to distinguish between the two groups (Fig. 2a), including one feature from T1-contrast-MSDE images, four from T1-weighted-MSDE images, six from T2-weighted-MSDE images, and two from T1-contrast images. Then, the coefficients of these features were used to develop the RF signature model. Receiver operating characteristic curve analysis was performed to evaluate the ability of the LASSO model to predict response to different treatments. In the training group, the optimal cutoff of the signature model was − 100.4, sensitivity was 97.8%, and specificity was 93.8% (Fig. 2b). In the validation group, we used − 100.4 as the cutoff value, which yielded sensitivity, specificity, and area under the curve values of 83.3%, 93.7%, and 97.7%, respectively (Fig. 2c).

To study the relationship between the RF signature model and clinical characteristics, we arranged patients in the training group in ascending order of the RF signature in the heatmap. We found that the signature exhibited a close relationship with the number of days from symptom onset, history of dyslipidemia, smoking, fibrin degradation product (FDR) levels, and D-dimer levels (Fig. 2d). Signature values significantly increased in patients with dyslipidemia and those with a history of smoking (Fig. 2e, f). In addition, when the median value of the signature was used to stratify patients into high-score and low-score groups, FDR and D-dimer levels were significantly higher in the high-score group than in the low-score group. In contrast, symptom duration tended to be shorter in the high-score group than in the low-score group (Fig. 2g–i). These results indicated that the radiomics features can also describe the clinical characteristics that are closely related to thrombosis formation. That is the reason the signature model performed that well in clarifying patients’ responses to drug treatment and selecting patients for EVT.

### Performance of the CVST-SVM model

**Fig. 3:**
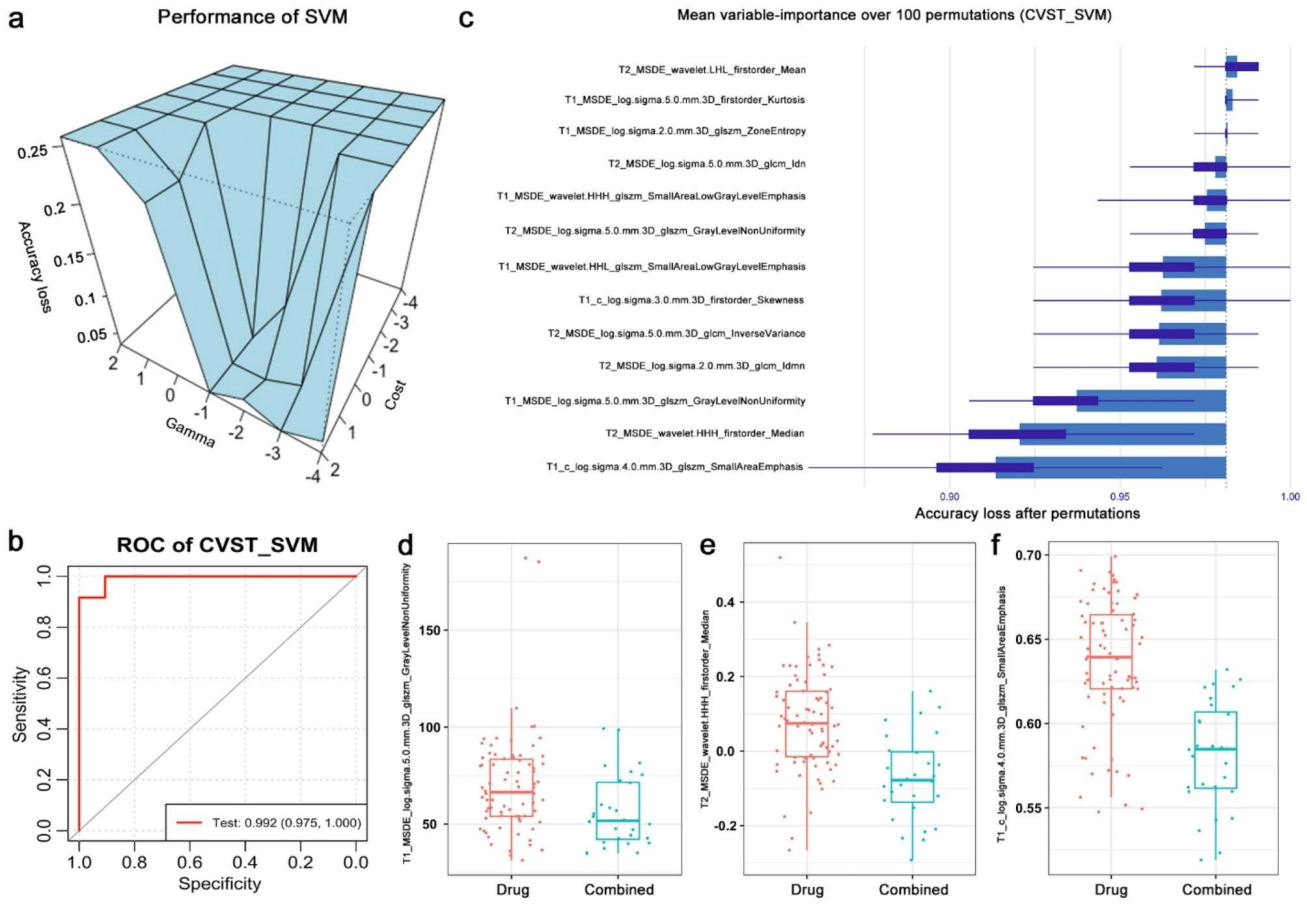
Performance of the CVST-SVM model. (**a**) The accuracy loss, gamma, and cost value of the CVST-SVM model. (**b**) The permutations of the CVST-SVM model. (**c**) The receiver operating characteristic (ROC) curve of the CVST-SVM model in the validation group. (**d**–**f**) The top three features in the anticoagulant-only and combined treatment groups. CVST: cerebral venous sinus thrombosis; SVM: support vector machine

The 13 features selected were used to create the CVST-SVM model (Fig. 3a). In the training set, the sensitivity and specificity of the CVST-SVM model reached 93.8% and 100%, respectively (**Online Resource 1**). In the validation group, the sensitivity and specificity of the CVST-SVM model reached 91.7% and 96.9%, respectively (Fig. 3b and **Online Resource 1**).

**Fig. 4:**
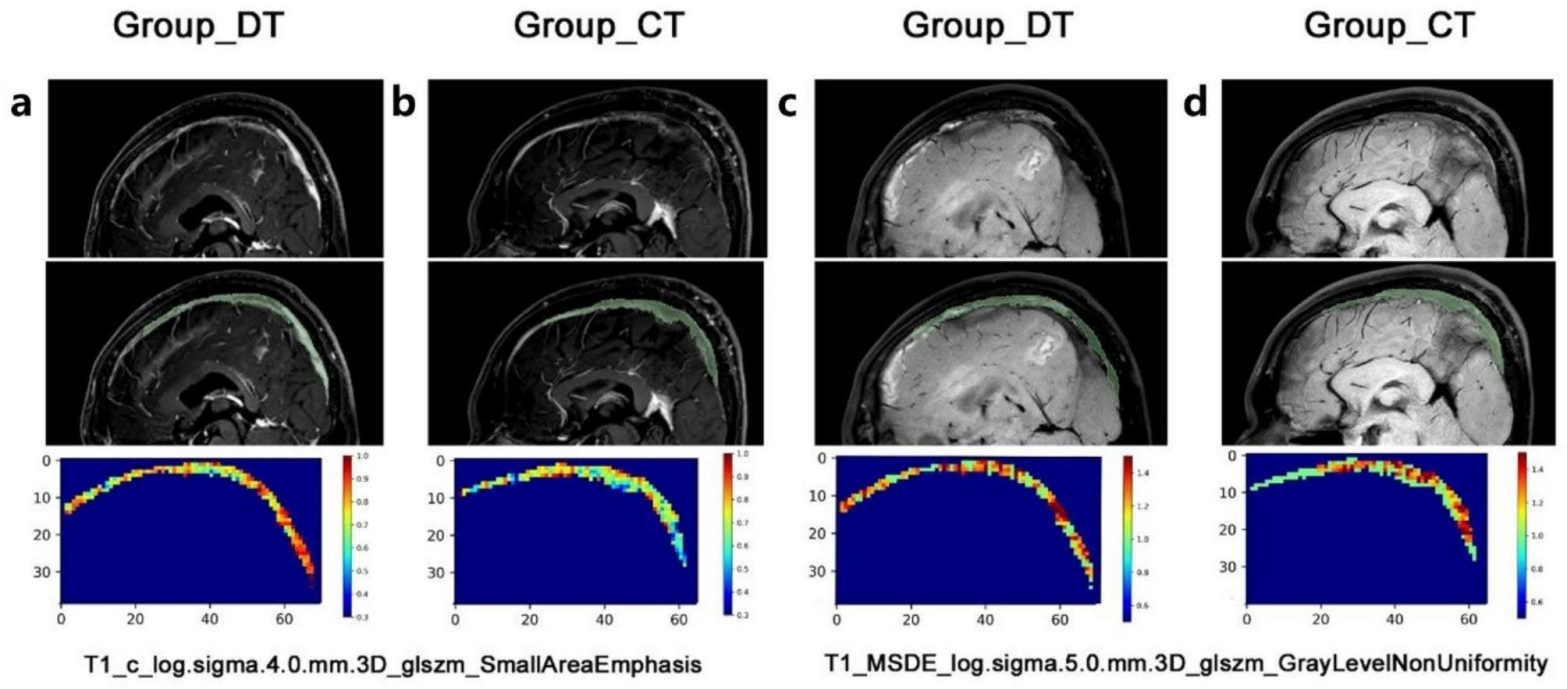
The representative image. The original image (first row), region of interest (second raw), and the main different radiomic features (third row) of T1_c_log.sigma.4.0.mm.3D_glszm_SmallAreaEmphasis(**a-b**) and T1_MSDE_log.sigma.5.0.mm.3D_glszm_GrayLevelNonUniformity(**c-d**) in the anticoagulant-only and combined treatment groups

In addition, we used the “permutation method” to list the importance of each feature in the CVST-SVM model (Fig. 3c). The top three important features identified were “log.sigma.5.0. mm.3D.glszm.GrayLevelNonUniformity” in the T1-weighted-MSDE image (0.639 vs. 0.585, *p* < 0.001), “wavelet.HHH.firstorder.Median” in the T2-weighted-MSDE image (0.074 vs. −0.078, *p* < 0.001), and “log.sigma.4.0.mm.3D.glszm.SmallAreaEmphasis” in the T1-contrast image (66.410 vs. 51.654, *p* = 0.004). These features were significantly compared with the data from the combined group (Fig. 3d–f). To illustrate how the CVST-SVM model functions, we plotted the “GrayLevelNonUniformity feature” and “SmallAreaEmphasis” in a voxel-wise manner (Fig. 4a-d).

.

## Discussion

In this study, we created two machine learning models based on HRMRI to predict the response of patients with CVST to anticoagulation therapy, and both models could screen endovascular candidates who could not recover with drug treatment alone.

### The treatment of CVST and the dilemmas that need to be solved

The first-line treatment for CVST is anticoagulation therapy with subcutaneous LMWH [1]. Dabigatran and warfarin can be further administered orally to prevent the recurrence of venous thrombotic events. According to current expert consensus, mechanical thrombectomy is considered when anticoagulation fails or if symptoms continue to worsen [10]. This is because the duration of severe CVST is closely associated with parenchymal damage [14]. Prolonged venous sinus occlusion can damage the brain parenchyma, which increases the risk of hemorrhage [15] and/or infarction [16] in the parenchyma. Thus, timely recanalization of blood vessels is of great clinical benefit. Mechanical thrombectomy has been found to be more effective in removing long-lasting thrombi and those involving multiple sinuses. The current results suggest that combining thrombolysis and mechanical thrombectomy can benefit many patients with CVST [17].

The appropriate timing for the initiation of mechanical thrombectomy remains controversial. In a previous study, the indication for EVT was mostly based on mRS scores, which are closely associated with clinical manifestations. In addition, the Society of Neurointerventional Surgery recommends mechanical thrombectomy for patients who tend to deteriorate during anticoagulation therapy and those with severe neurological deficits or coma [5]. However, the clinical manifestations of CVST are greatly variable, and clinical symptoms alone are not sufficient for predicting the outcomes of interventional therapies [16]. In the current study, we observed no significant differences in the rate of intracranial hemorrhage or venous infarction between the groups. In addition, even after accounting for clinical characteristics such as sex, age, medical history, and coagulation function, we failed to observe differences between the treatment groups, emphasizing the importance of developing additional models to accurately classify patients who may require mechanical thrombectomy.

### The application of radiomics provides more subtle features

Radiological examination, especially MRI, plays an important role in evaluating the nature of the thrombus in patients with CVST [18]. Standard spin-echo T1/T2 MRI has advantages in defining the stages of thrombus evolution since different signals can reflect different stages of hemoglobin degradation [19]. Using magnetic resonance venography or contrast-contrast MR venography can increase the sensitivity of the abnormal signal for diagnosing suspected CVST [20]. Recently, black-blood contrast has been used to make imaging-based diagnosis of CVST more accurate by suppressing the blood signal in the venous sinus lumen. In a 5-year study of real-world clinical practice, black-blood images were shown to improve diagnostic confidence in defining the state of CVST [21]. Further studies have suggested that in addition to defining the characteristics of the clot, black-blood images may be useful in predicting complete recanalization [22, 23]. In our study, we evaluated all RFs instead of focusing solely on acute clot features along when building our models. This approach is particularly advantageous given that radiomic methods can extract subtle features that may not be easily perceived by the human eye [24, 25].

### The benefits of HRMRI-based radiomics in analyzing venous sinus related diseases

Previous studies have demonstrated that using a radiomic model provides a strong advantage in the analysis of venous sinus-related diseases, especially idiopathic intracranial hypertension (IIH). Considering that IIH is always caused by stenosis of the venous sinus [26] and that the pressure of such stenosis is positively correlated with failure risk in patients undergoing transverse sinus stenting [27], RFs were successfully used to predict the prognosis of IIH based on trans-stenotic pressure and arteriography-derived hemodynamic features [28, 29]. The current findings support the use of RF-based models for predicting EVT suitability in patients with CVST.

Multiple RFs are used to characterize the shape and heterogeneity of a lesion, making them essential for evaluating thrombosis [30, 31]. Variations in shape and heterogeneity reflect the different portions of thrombosis, leading to more detailed differentiation of thrombus characteristics. A previous study used radiomic-based thrombus features to predict recanalization outcomes in patients with acute ischemic stroke and included 67 patients with internal carotid or middle cerebral artery segment thrombi and used 326 predefined RFs to describe thrombus characteristics. The study found that combining all RFs in a statistical model led to a more promising predictive value for recanalization than the use of thrombus imaging biomarkers such as the length and permeability of the thrombus [25]. Thus, radiomic analysis may aid in delineating the characteristics of the thrombus that can be used to stratify patients suitable for EVT.

In our study, machine learning helped identify the most accurate models, which included 13 RFs. Among these RFs, “T1_MSDE_ log. sigma.5.0. mm.3D_glszm_GrayLevelNonUniformity,” “T2_MSDE_wavelet.HHH_firstorder_Median,” and “T1_c_ log.sigma.4.0.mm.3D_glszm_SmallAreaEmphasis” were the most important features. The gray-level non-uniformity in the GLSZM reflects the variability of gray-level intensity values. The T1-MSDE sequence describes the interwall and intraluminal well [32]. A high gray-level non-uniformity value indicates more lumina and loose connections between the thrombosis and vessel wall, which creates more space for drug penetration. Consequently, these patients are more likely to benefit from drug treatment. In addition, T1-contrast images were used to define the characteristics of thrombosis and remaining blood [33]. Lower values for small zones indicate coarser textures, suggesting a relatively higher level of fibrosis within the thrombosis. Therefore, these patients are likely to require mechanical thrombectomy given that drug treatment may not be effective. In summary, these results demonstrate that RF-based models are both practical and sensitive to small but meaningful characteristics of thrombosis.

This study had some limitations. First, this was a single-center retrospective study with a relatively small sample size. Further studies are required to verify the robustness of our model in larger sample sizes of patients from multiple centers. Second, two patients with failed recanalization were excluded from the combined treatment group, and it is possible that their thromboses shared radiomic characteristics with successful recanalization. However, predicting successful EVT was not the focus of this study. Lastly, the manual delineation of ROIs in this study may have resulted in inter-rater variability, which could affect the reproducibility and generalizability of the results. To address this issue, future studies should utilize automated segmentation methods using U-net-based deep learning techniques.

## Conclusions

The CVST-SVM model built based on RFs extracted from HRMRI exhibited favorable sensitivity and specificity for selecting EVT candidates among patients with CVST. The early introduction of EVT in the treatment course using the radiomic model proposed in this study may potentially benefit patients with CVST.

### Electronic supplementary material

Below is the link to the electronic supplementary material.

Supplementary Material 1

## Data Availability

Relevant data and materials are available through contacting the corresponding authors.
