# Supplementary material for: High-resolution magnetic resonance imaging-based radiomic features aid in selecting endovascular candidates among patients with cerebral venous sinus thrombosis
Source: Thromb J. 2023 Nov 10;21:116. doi: 10.1186/s12959-023-00558-4 (PMC10636961; doi:10.1186/s12959-023-00558-4)
Supplement: Supplementary file 1 — Supplementary Material 1 [file 12959_2023_558_MOESM1_ESM.pdf]

Title: High-resolution magnetic resonance imaging-based radiomic features aid in selecting endovascular candidates among patients with cerebral venous sinus thrombosis

Journal: Translational Stroke Research

Author Names: Yu-Zhou Chang, Hao-Yu Zhu, Yu-Qi Song, Xu Tong, Xiao-Qing Li, Yi-Long Wang, Ke-Hui Dong, Chu-Han Jiang, Yu-Peng Zhang, Da-Peng Mo

Corresponding Author: Da-Peng Mo, MD; Department of Interventional Neuroradiology, Beijing Neurosurgical Institute, Beijing Tiantan Hospital, Capital Medical University, No. 119 South 4th Ring West Road, Fengtai District, Beijing 100070, P.R. China Tel: (8610) 59978857, Email: bjttmodp@163.com

Online Resource 1. Model performance metrics of CVST_SVM

**Metrics**	**Training**	**Validation**
**Sensitivity**	**0.938**	**0.917**
**Specificity**	**1**	**0.969**
**Accuracy**	**0.984**	**0.955**
**F1 score**	**0.968**	**0.917**

CVST, cerebral venous sinus thrombosis; SVM, support vector machine
